# Identification of novel drug targets through integrative PWAS of brain and plasma proteins with Ulcerative Colitis GWAS

**DOI:** 10.1371/journal.pone.0324035

**Published:** 2025-05-30

**Authors:** Ningning Liu, Yi Hou, Miao Yu, Gaihong Liu, Yingxue Xu, Qiang Jiang, Dongli Wang, Lianzhu Wang, Yujie Zhao

**Affiliations:** 1 Affiliated Hospital of Shandong University of Traditional Chinese Medicine, Jinan, Shandong, China; 2 Shandong University of Traditional Chinese Medicine, Jinan, Shandong, China; 3 The Third People’s Hospital of Jinan City, Jinan, Shandong, China; International Medical University, MALAYSIA

## Abstract

Previous genome-wide association studies (GWAS) have identified various risk variants for ulcerative colitis (UC), but there is a lack of evidence showing how these variants contribute to the development of UC. We employed an integrated pipeline to effectively translate genetic associations in order to identify pathogenic genes for UC.By combining GWAS data for UC with proteomic data from the human brain and plasma, we conducted a protein-wide association study (PWAS) and utilized protein-protein interaction (PPI) network analysis to screen for potential key proteins. Subsequently, causal analysis was performed to assess the potential causal relationships between these proteins and the risk of developing UC.Multiple genes associated with UC were identified in the human brain and plasma proteomes, including known genes such as TYK2 and STAT3, as well as newly discovered genes such as NARS2. PPI networks revealed strong interactions among proteins, including TYK2, STAT3, and IL23R. Causal analysis indicated that 11 risk genes, including FCGR2A, showed significant causal associations with UC, and were linked to key processes related to immune regulation and inflammatory responses, suggesting their potential roles in the pathogenesis of UC.This study integrated GWAS and PWAS data to identify risk genes associated with UC, providing new insights into the disease’s pathogenesis and potential therapeutic targets.

## 1. Introduction

Ulcerative colitis (UC) is an idiopathic chronic inflammatory disease of the colonic mucosa that begins in the rectum and typically extends proximally in a continuous manner through part or all of the colon [1]. The onset of UC is a complex pathological process influenced by multiple factors, including genetic, environmental, and immune factors. Family history is recognized as the most significant independent risk factor [2]. Previous large-scale genome-wide association studies (GWAS) have identified numerous genetic variations associated with inflammatory bowel disease (IBD), determining 38 susceptibility loci for IBD [3]. Moreover, patients with UC often experience alterations in mental status, suggesting a close relationship between UC and the gut-brain axis [4].

Proteins are the most effective biomarkers and therapeutic targets [5,6], as they represent key functional components of cellular and biological processes and are the ultimate products of gene expression [7]. Previous studies have indicated significant differences in the protein expression profiles of UC tissues compared to normal controls, suggesting that the pathogenesis of UC may be associated with various proteins [8]. By conducting in-depth studies on proteins, we aim to reveal the genetic basis of UC more comprehensively, which could provide new insights and targets for the diagnosis, treatment, and prevention of UC.

Protein-wide association studies (PWAS) integrate GWAS data with proteomic data to identify candidate genes associated with specific traits [9]. In this study, we combined GWAS data for UC with PWAS data from human brain and plasma proteomes to identify risk genes related to UC in association with brain and blood proteomes. Our findings offer valuable insights into the potential biological mechanisms underlying the development of UC related to these genes.

## 2. Materials and methods

### 2.1 Data sources

#### GWAS summary statistics.

The GWAS summary data used the largest UC GWAS ever published, including a total of 412,561 European control and 5371 UC patients from UKB [10]. The genotypes were further imputed using a combination of the Haplotype Reference Consortium, UK10K, and 1000 Genomes Project Phase 3 reference panels by IMPUTE4 software [11]. We excluded the variants with (1) an imputation information metric (INFO score) ≤ 0.8; (2) MAF ≤ 0.0001 (except for missense and protein-truncating variants annotated by VEP51, which were excluded if MAF ≤ 1 × 10 − 6); and (3) PHWE ≤ 1 × 10 − 10, resulting in 13,791,467 variants analyzed in total.

#### Brain proteomic and genetic data.

We utilized two independently published datasets on human dorsolateral prefrontal cortex (dPFC) proteins and genotypes from the Religious Order Study and Rush Memory and Aging Project (ROS/MAP) [12] and the Banner Sun Health Research Institute (Banner) [13]. The proteomic analysis was conducted with isobaric tandem mass tag peptide labeling, followed by examination through liquid chromatography-mass spectrometry. Participants in the ROS/MAP cohort underwent genotyping via whole-genome sequencing or genome-wide genotyping using the Illumina OmniQuad Express or Affymetrix GeneChip 6.0 platforms, whereas those in the Banner cohort were genotyped with the Affymetrix Precision Medicine Array. Following quality control measures, the PWAS encompassed 8,356 proteins from 376 individuals within the ROS/MAP dataset and 8,168 proteins from 152 individuals within the Banner dataset.

#### Blood proteomic and genetic data.

The blood proteomics data alongside genotyping information were sourced from the Atherosclerosis Risk in Communities (ARIC) study. The plasma protein or protein complex concentrations in the study were quantified using the slow off-rate modified aptamer (SOMAmer) technology, which serves as a platform for proteomic profiling. Genotyping was carried out using the Affymetrix 6.0 DNA microarray. Post quality control, the analysis included a total of 4,483 distinct proteins from 7,213 European participants in the ARIC study.

#### Ethical Statement.

This research exclusively analyzed publicly available data from existing databases, with no new data collection or direct interaction with human participants. Consequently, ethical approval and informed consent were not applicable.

### 2.2 Statistical approach

#### PWAS.

We used FUSION standard pipeline [14] to integrate brain protein data and GWAS summary statistics for discovery PWAS analysis. The SNP effect values in GWAS were weighted by the weight of SNP in the protein model data and then aggregated to calculate the effect of the target protein on the disease. To control the potential effect of multiple testing on the study results, the false discovery rate (FDR) of *P* < 0.05 was used as the significance threshold in our PWAS analysis. Plasma proteins were then analyzed using the same procedure to identify UC risk proteins in plasma (FDR of *P* < 0.05).

#### Causal analysis.

To determine causal relationships from our PWAS findings, we utilized two independent methods. For Bayesian colocalization analysis [15], we used the COLOC tool within the FUSION software to estimate the posterior probability that the same variant affects both GWAS and pQTL signals. Under this framework, five hypotheses (H0 to H4) were evaluated, with H4 suggesting a shared causal SNP. Causality was established if the posterior probability for H4 exceeded 0.5. To further validate these relationships, we applied the SMR method [16], using pQTL data and UC GWAS data. Significant causal associations were confirmed with SMR and HEIDI test (the FDR-corrected *P*-value of SMR < 0.05 and unadjusted *P*-value of HEIDI > 0.05).

#### Protein-protein interaction.

To investigate causal genes involved in three diseases, we conducted an extensive network analysis using the STRING database [17]. We input 14 UC-related proteins identified in brain and blood PWAS into the website, retaining edges with an interaction score higher than 0.4.

## 3. Results

### 3.1 Discovery and Replication of PWAS in UC

**Fig 1:**
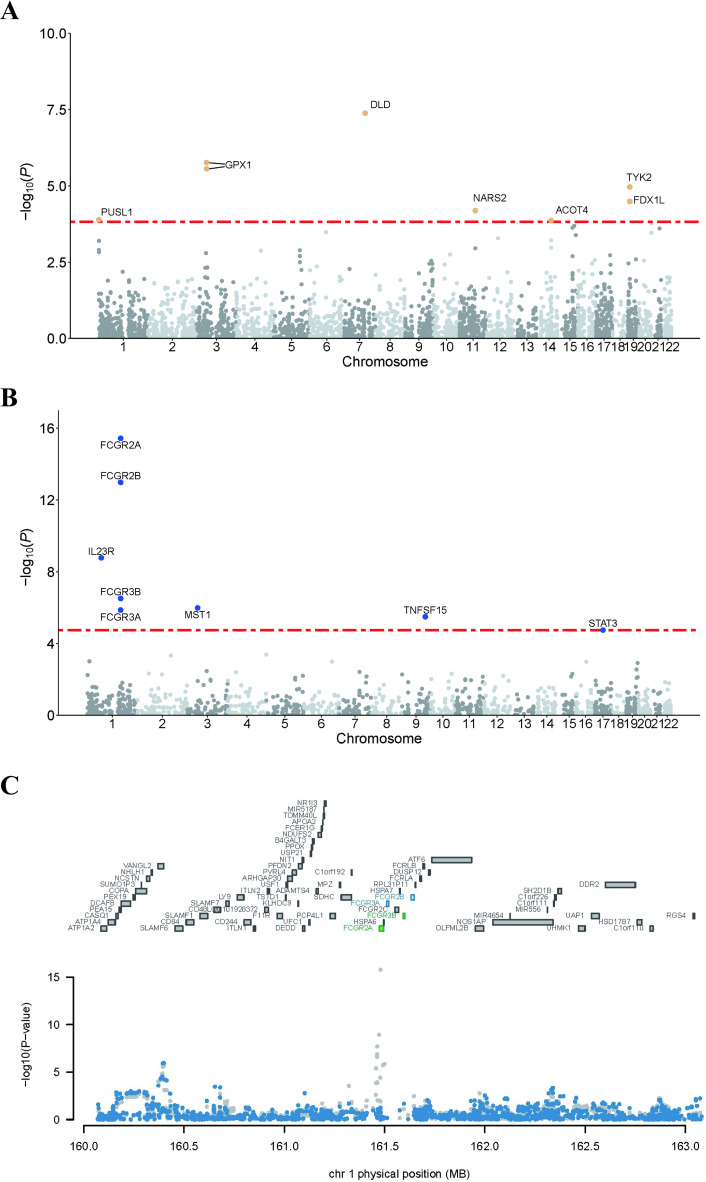
Manhattan plots of PWAS results in brain tissue and blood, and conditional association analysis.

To identify brain proteins associated with UC susceptibility, we performed PWAS and FUSION analyses using brain proteomes from the ROS/MAP and Banner cohorts. After quality control, the proteomic profiles included 8,356 proteins from ROS/MAP and 8,168 proteins from Banner, of which 2,608 proteins (1,469 from ROS/MAP and 1,139 from Banner) exhibited significant heritability (*P *< 0.01) and were included in the PWAS analysis. Out of these 2,608 associations, the brain PWAS identified cis-regulated brain protein levels associated with UC for 7 distinct genes, with an FDR of *P *< 0.05 (Fig 1A and Table 1). Among these, 6 proteins were significantly negatively associated with UC (DLD, GPX1, TYK2, FDX1L, NARS2, PUSL1), while 1 protein (ACOT4) was significantly positively associated with UC.

**Table 1:** The discovery of brain PWAS identified 8 significant genes.

PANEL	ID	CHR	P0	P1	PWAS.Z	PWAS.P	P.FDR
ROSMAP	DLD	7	107531415	107572175	-5.49	4.13 × 10-8	1.19 × 10-4
ROSMAP	GPX1	3	49394609	49396033	-4.78	1.73 × 10-6	0.003
BANNER	GPX1	3	49394609	49396033	-4.69	2.75 × 10-6	0.003
BANNER	TYK2	19	10461209	10491352	-4.40	1.08 × 10-5	0.008
ROSMAP	FDX1L	19	10416103	10426691	-4.15	3.25 × 10-5	0.019
BANNER	NARS2	11	78147007	78285919	-4.00	6.47 × 10-5	0.031
BANNER	PUSL1	1	1243947	1247057	-3.83	1.28 × 10-4	0.049
BANNER	ACOT4	14	74058410	74063200	3.85	1.36 × 10-4	0.049

The plasma PWAS identified cis-regulated plasma protein levels associated with UC for 8 distinct genes, with an FDR of *P* < 0.05 (Fig 1B and Table 2). Of these, 7 proteins were significantly negatively associated with UC, while 1 protein was significantly positively associated. Four proteins (FCGR2A, FCGR2B, MST1, TNFSF15) were significantly negatively associated with UC, while four proteins (IL23R, FCGR3B, FCGR3A, STAT3) were significantly positively associated with UC.

**Table 2:** The discovery of blood PWAS identified 8 significant genes.

ID	CHR	P0	P1	PWAS.Z	PWAS.P	P.FDR
FCGR2A	1	161475220	161493803	-8.15	3.69 × 10-16	4.80 × 10-13
FCGR2B	1	161632937	161648444	-7.43	1.05 × 10-13	6.84 × 10-11
IL23R	1	67604590	67725662	6.03	1.67 × 10-9	7.25 × 10-7
FCGR3B	1	161592986	161601753	5.12	3.09 × 10-7	1.01 × 10-4
MST1	3	49721380	49726934	-4.88	1.04 × 10-6	2.71 × 10-4
FCGR3A	1	161511549	161520527	4.83	1.36 × 10-6	2.95 × 10-4
TNFSF15	9	117546932	117568319	-4.66	3.21 × 10-6	5.97 × 10-4
STAT3	17	40465342	40540558	4.29	1.77 × 10-5	0.003

The plasma PWAS identified that four proteins (FCGR2A, FCGR2B, FCGR3B, and FCGR3A) were tightly clustered in the 161.5–162 Mb region of chromosome 1. We conducted conditional analyses of these proteins, as shown in Fig 1C. Through these analyses, we found that the significance of FCGR2B and FCGR3A was driven by joint significance due to associations with other genes, and thus we did not perform subsequent causal analyses.

Panels A-B depict the PWAS results for brain tissue and plasma, while panel C illustrates the conditional association analysis of PWAS results in blood. Each point represents a single association test between a gene and UC, ordered by genomic position on the x-axis and strength of association on the y-axis, as the -log10(P) of the z-score test. PWAS identified 15 genes with cis-regulated brain protein abundance associated with UC, with FDR *P *< 0.05. The red horizontal line denotes the FDR significance threshold of *P *< 0.05, set at the highest unadjusted P value below the threshold (*P *= 1.4 × 10⁻⁴).

### 3.2 Protein-Protein Interaction Networks in UC

**Fig 2:**
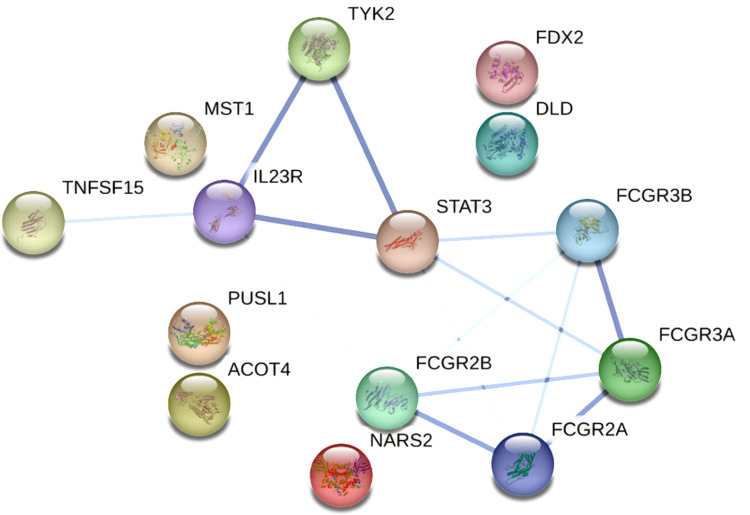
PPI network and pathways among the 14 significant proteins identified in PWAS for UC.

We utilized the STRING database to investigate the connectivity among the 14 UC-associated proteins identified from PWAS, revealing a protein community based on protein-protein interactions (PPIs). A community refers to a group of proteins that are more closely connected to each other than to proteins in other groups. The community includes TNFSF15, TYK2, STAT3, IL23R, FCGR2B, FCGR3B, FCGR2A, and FCGR3A (Fig 2), comprising one brain protein and seven plasma proteins. This suggests that TYK2 may be a key protein involved in the blood-brain barrier mechanism.

Different line colors represent distinct types of interactions. Pathway enrichment was determined using a hypergeometric test with Bonferroni correction for multiple testing.

### 3.3 Causal Analysis Results of Genomics and Proteomics

To further evaluate whether cis-regulated brain protein expression mediates the association between genetic variants of the 15 genes and UC, we applied COLOC and SMR analyses on the same discovery dataset [16]. Multiple genes showed significant colocalization and causal associations (Tables 3-4).

The COLOC analysis indicated that 10 genes, including FCGR2A, showed significantly high colocalization probabilities. The SMR analysis revealed that 7 genes, including FCGR2A, had significant causal relationships (*P *< 0.05). Subsequently, we conducted a heterogeneity test using the HEIDI tool [16] to distinguish between pleiotropic/causal and linkage relationships for these 7 genes. The HEIDI results suggested that 4 out of the 7 genes might be significantly affected by linkage disequilibrium, while 3 genes were consistent with pleiotropy or causal relationships (Tables 3-4). Both SMR and HEIDI analyses indicated that 3 genes (IL23R, DLD, and GPX1) may be related to UC through cis-regulated brain protein abundance (Tables 3-4). The integrated analysis of COLOC and SMR demonstrated that 11 risk genes, including FCGR2A, have significant causal associations with UC.

**Table 3:** Colocalization and causal analysis results for blood genes and the target trait.

ID	CHR	P0	P1	PWAS.Z	PWAS.P	COLOC.PP4	P.SMR	P.HEIDI	Causal
FCGR2A	1	1.61E + 08	1.61E + 08	-8.14846	3.69E-16	1	2.27E-16	1.80E-15	YES
IL23R	1	67604590	67725662	6.02728	1.67E-09	0.998	6.33E-02	6.11E-02	YES
FCGR3B	1	1.62E + 08	1.62E + 08	5.11762	3.09E-07	0.992	7.44E-07	6.16E-03	YES
MST1	3	49721380	49726934	-4.8841	1.04E-06	0.994	7.50E-07	6.73E-03	YES
TNFSF15	9	1.18E + 08	1.18E + 08	-4.6568	3.21E-06	0.074	7.06E-05	1.34E-01	YES
STAT3	17	40465342	40540558	4.292	1.77E-05	0.909	2.39E-05	8.34E-06	YES

**Table 4:** Colocalization and causal analysis results for brain genes and the target trait.

ID	CHR	P0	P1	PWAS.Z	PWAS.P	COLOC.PP4	P.SMR	P.HEIDI	Causal
DLD	7	1.08E + 08	1.08E + 08	-5.48513	4.13E-08	0.982	1.39E-04	1.86E-01	YES
GPX1	3	49394609	49396033	-4.78319	1.73E-06	0.974	7.52E-06	9.96E-01	YES
FDX1L	19	10416103	10426691	-4.1549	3.25E-05	0.984	–	–	YES
GPX1	3	49394609	49396033	-4.689	2.75E-06	0.977	–	–	YES
TYK2	19	10461209	10491352	-4.40121	1.08E-05	0.983	–	–	YES
NARS2	11	78147007	78285919	-3.995	6.47E-05	0.147	–	–	NO
PUSL1	1	1243947	1247057	-3.83	0.000128	0.024	–	–	NO
ACOT4	14	74058410	74063200	3.81605	0.000136	0.089	–	–	NO

### 3.4 Drug-target analysis

Given the robust results demonstrating the associations between causal proteins and UC, we further explored drug repurposing opportunities for these proteins by consulting DrugBank. Among 15 UC-associated proteins, 8 serve as drug targets involving 54 different drugs. Notably, Tofacitinib, which targets TYK2, has been clinically approved for treating UC by mitigating inflammatory responses through the inhibition of the JAK-STAT signaling pathway. Besides Tofacitinib, other medications such as Etanercept have also been utilized in the treatment of immune-mediated or inflammatory diseases. Our research provides a novel genetic foundation for these drugs in the context of UC treatment and may facilitate further investigations into developing additional targeted therapies for UC (Supplementary Table S1).

## 4. Discussion

UC is a chronic, relapsing gastrointestinal inflammatory disease with a complex genetic and environmental etiology. To identify genetic variations contributing to UC risk, we employed a series of analytical techniques to investigate the functional associations between protein biomarkers in the brain and UC. We identified 15 candidate risk genes associated with altered protein abundance in both brain and plasma in UC (DLD, GPX1, TYK2, FDX1L, NARS2, PUSL1, ACOT4, FCGR2A, FCGR2B, IL23R, FCGR3B, MST1, FCGR3A, TNFSF15, STAT3). Furthermore, through colocalization and causal inference in brain and PWAS related to UC, we identified 11 candidate risk genes (FCGR2A, IL23R, FCGR3B, MST1, TNFSF15, STAT3, DLD, GPX1, FDX1L, GPX1, TYK2) with causal associations to UC. These genes have the potential to elucidate the molecular mechanisms underlying UC development and provide a theoretical foundation for the development of novel therapeutic strategies. Identifying genetic targets influencing UC is crucial for uncovering the disease’s pathogenesis and developing innovative therapeutic agents.

Our analysis focuses on previously studied susceptibility genes in UC. TYK2 is a widely expressed tyrosine kinase that mediates signaling for immune-regulatory cytokines and acts on various immune and non-immune cells [18]. It plays a crucial role in several immune processes, including natural killer (NK) cell activity, B cell maturation, and the differentiation of Th1 and Th17 cells [19]. TYK2 is a key signaling kinase in the JAK-STAT pathway, particularly relevant to the pro-inflammatory receptors for cytokines such as IL-23, and is central to the pathogenesis of inflammatory bowel diseases, including UC. The IL-23/IL-12 complex binds to the IL-12Rβ1/IL-23R receptors on Th17 cells, activating TYK2/JAK2, which leads to the phosphorylation of STAT3. Phosphorylated STAT3 then dimerizes, translocates, and binds to DNA, resulting in the production and release of inflammatory factors IL-17, IL-21, and IL-22, collectively influencing inflammatory bowel diseases like UC [20]. These findings align with our study results. The results from PPI analysis further demonstrate a strong interaction among TYK2, STAT3, and IL23R. Additionally, prior studies have identified DLD, GPX1, FDX1L, TNFSF15, FCGR2A, FCGR3B, FCGR3A, and MST1 as susceptibility genes for UC [21–26]. These findings are consistent with our results.

In the results of the PWAS, we observed that the genes FCGR2A, FCGR2B, FCGR3B, and FCGR3A are closely clustered in the 161.5–162 Mb region of chromosome 1. All these genes belong to the Fcγ receptor family and are expressed by a variety of innate and adaptive immune cells, mediating inflammatory responses through binding to the Fc portion of immunoglobulin G, which is associated with systemic autoimmune diseases [27]. The FCGR genes exhibit high homology, and dysregulation of Fcγ receptors plays a critical role in various inflammatory diseases, including rheumatoid arthritis (RA) and systemic lupus erythematosus (SLE) [28–30]. Furthermore, previous studies have shown that copy number variations or single nucleotide polymorphisms of these genes are closely linked to autoimmune diseases [31]. Overall, the clustered distribution and functional similarities of these genes suggest they may collectively participate in biological processes such as immune responses and inflammatory reactions. However, in our subsequent causal analysis, only FCGR2A and FCGR3B exhibited significant causal associations, indicating that these two genes may play a direct role in the pathogenesis of UC, while the associations of FCGR2B and FCGR3A may be indirect or false positives. Previous research has found a significant association between FCGR3A and UC susceptibility [26], while the correlation of FCGR2B in UC patients is relatively weaker [32]. In our analysis, the newly identified susceptibility genes include NARS2, PUSL1, ACOT4, and FCGR2B. These newly identified genes provide new research leads, suggesting they may play important roles in the pathogenesis of UC. NARS2 is a putative member of the class II family of aminoacyl-tRNA synthetases, playing a crucial role in protein biosynthesis and serving as a major effector in the inflammatory responses of RA and SLE [33]. Therefore, it is likely that it is also significantly associated with UC.

Our study has several strengths. First, we conducted the largest and most comprehensive PWAS of UC to date, based on the latest summary statistics from GWAS related to UC. Second, we analyzed a core protein module consisting of eight proteins, including TNFSF15, using PPIs, which further revealed the biological connections among the proteins. Additionally, through causal analysis, we identified the more significant genes involved, unveiling potential causal genes related to the pathogenesis of UC.

However, there are several limitations to the current study. First, pQTL and eQTL mapping cannot resolve all GWAS signals. At the single level, such as the protein level, it is challenging to interpret the functions of genes in the biological development of UC. Further epigenetic studies based on mQTL, single-cell sequencing, and whole-genome sequencing are needed to design tailored therapeutic strategies and provide a comprehensive understanding of the molecular mechanisms related to UC [34,35]. Moreover, given the ethnic variability in the current proteomic samples and the relatively small sample size, expanding the scale and diversity of brain proteomic data would aid in making more accurate estimates and facilitating broader applications.

## Supporting information

Table S1 Results of the Drugbank annotated for the UC-associated genes. (XLSX)
